# An Otogenic Variant of Lemierre's Syndrome Caused by Trueperella bernardiae: A Case Report and Literature Review

**DOI:** 10.7759/cureus.42977

**Published:** 2023-08-05

**Authors:** Takumi Kumai, Takahiro Inoue, Shota Sakaue, Kenzo Ohara, Miki Takahara

**Affiliations:** 1 Department of Otolaryngology, Head and Neck Surgery, Asahikawa Medical University, Asahikawa, JPN

**Keywords:** septic pulmonary embolism, meningitis, trueperella bernardiae, lemierre syndrome, chronic otitis media

## Abstract

Upper airway infections caused by anaerobic bacteria, including pharyngitis and tonsillitis, are a common cause of septic thrombosis (Lemierre's syndrome). Although otitis media rarely progresses to systemic infection, an abscess surrounding the middle ear can affect the central nervous system. *Trueperella bernardiae* was originally considered a non-pathogenic aerobic bacterium but has subsequently been reported to cause bacteremia and brain abscesses. Here, we report a case of otitis media caused by *T. bernardiae* complicated by meningitis, subdural empyema, and septic pulmonary emboli in an immunocompetent patient.

## Introduction

The oral cavity and pharynx are major gateways for bacteria. Because the internal jugular vein is in close proximity to the pharynx, upper airway infections, including pharyngitis and tonsillitis, can cause internal jugular vein thrombosis, followed by septic pulmonary emboli, known as Lemierre's syndrome. Although the incidence of Lemierre's syndrome has decreased since the advent of antibiotics, upper airway infections with “red flag” symptoms are still a life-threatening disease.

Oropharyngeal infection is a typical cause of Lemierre's syndrome [1]. Because otitis media rarely leads to venous thrombosis, few reports have suggested that ear infections can induce a rare variant of Lemierre's syndrome [2]. Infection and inflammation from mastoiditis subsequent to otitis media can cause thrombosis in the sigmoid sinus instead of the internal jugular vein, a typical target in this syndrome. In most cases, anaerobic bacteria are considered pathogens. Trueperella bernardiae is a catalase-negative, Gram-positive aerobic bacillus that was assigned to Trueperella gen. nov. in 2011. Originally considered an opportunistic pathogen, severe infections, including sepsis and brain abscesses, with this bacterium have been reported [3,4]. Herein, we report a case of an otogenic variant of Lemierre’s syndrome caused by T. bernardiae in an immunocompetent patient.

## Case presentation

**Figure 1:**
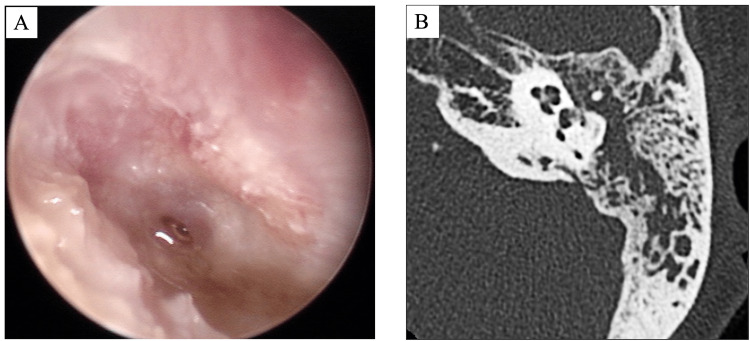
Images of the otitis media and mastoiditis prior to treatment (A) Flexible endoscopy showing perforated left tympanic membrane with otorrhea. (B) Computed tomography scan of the left temporal bone showing soft-tissue density in the tympanic cavity and mastoid.

A 60-year-old woman was admitted to our emergency center. The patient had left otorrhea for six months and experienced headaches and fever four days prior to admission. The convulsions subsided spontaneously within an hour. The patient had a Glasgow Coma Scale score of 14. Although Kernig’s sign was negative, neck stiffness and jolt accentuation were observed. The patient’s temperature was 40.5°C, with a pulse rate of 130 bpm, blood pressure of 93/64 mmHg, and a respiration rate of 24/min on admission. The left tympanic membrane was perforated due to otorrhea (Figure 1A). Blood tests showed leukocytosis (white blood cell count: 17920/µL) and severe thrombocytopenia (15000/µL), with elevated C-reactive protein (13.9 mg/dL) and procalcitonin (121 ng/mL) levels. The fibrin degradation product and D-dimer levels were 11.4 µg/mL and 15 µg/mL, respectively. After a platelet transfusion, cerebrospinal fluid testing revealed a high number of white blood cells with neutrophil predominance and a low glucose level (5 mg/dL), indicating meningitis. However, the cerebrospinal fluid tested negative for Streptococcus pneumoniae antigen and herpes simplex virus, varicella zoster virus, and cytomegalovirus DNA. To evaluate the relationship between the patient’s otorrhea and meningitis, computed tomography (CT) and magnetic resonance imaging (MRI) were performed. CT revealed that the left tympanic and mastoid cavities were filled with soft tissue (Figure 1B).

**Figure 2:**
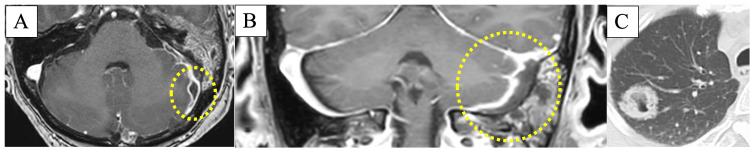
Images of the subdural empyema, sigmoid sinus thrombosis, and septic pulmonary emboli (A and B) Contrast-enhanced magnetic resonance imaging showing (A) subdural empyema and (B) sigmoid sinus thrombosis. (C) Computed tomography showing septic pulmonary emboli.

Although no bone defects were evident, contrast-enhanced MRI showed subdural empyema and sigmoid sinus thrombosis next to the left temporal bone (Figures 2A, 2B), indicating that the otitis media with mastoiditis directly involved local intracranial structures. Chest CT revealed a cavitary nodule with irregular walls in the superior lobe of the right lung (Figure 2C); however, tumor markers (neuron-specific enolase, cytokeratin fragment, and carcinoembryonic antigen) were negative.

**Figure 3:**
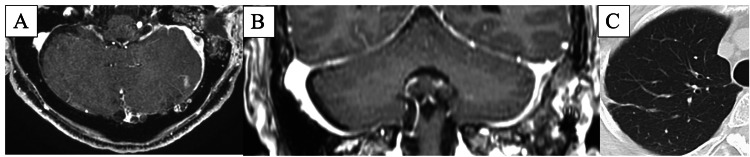
Follow-up imaging after treatment (A) Magnetic resonance imaging performed two months after treatment showing resolution of the subdural empyema. (B and C) Magnetic resonance imaging performed one year after treatment showing a reduction in the size of the thrombosis in sigmoid sinus (B) and the absence of septic pulmonary emboli (C).

The patient was diagnosed with left-sided otitis media with mastoiditis, subdural empyema, sigmoid sinus thrombosis, and sepsis-induced disseminated intravascular coagulation. Empiric antibiotic therapy with ampicillin (ABPC; 2 g every eight hours), vancomycin (VCM; 1.5 g, every 12 hours), and continuous heparin was initiated. Because of drug eruption, the ABPC was switched to meropenem (1 g every eight hours). The level of consciousness improved within a few days after antibiotic administration. T. bernardiae was isolated from blood cultures on day 11 of admission. The minimum inhibitory concentration (MIC) of ABPC, cefazolin (CEZ), cefmetazole (CMZ), cefotaxime (CXT), cefoxitin (FOX), imipenem (IPM), gentamycin (GM), arbekacin (ABK), erythromycin (EM), clindamycin (CLDM), minocycline (MINO), VCM, teicoplanin (TEIC), levofloxacin (LVFX), fosfomycin (FOM), linezolid (LZD), and sulfamethoxazole-trimethoprim (ST) was 0.12, 0.5, 1, 4, 0.5, 0.25, 4, 7, 0.12, 0.12, 2, 0.5, 0.5, 1, 32, 0.5, and 38 µg/mL, respectively. According to the European Committee on Antimicrobial Susceptibility Testing (EUCAST) pharmacokinetic-pharmacodynamic (PK/PD) non-species-related breakpoints, version 13.0, the isolate was susceptible to ABPC, CEZ, IPM, and LZD, had intermediate susceptibility to LVFX, and was resistant to CXT and GM. The platelet and white blood cell counts and C-reactive protein level recovered to normal by day 20. However, the white blood cell count and C-reactive protein level increased again on day 30. As the antimicrobial MIC was acceptable, meropenem was switched to LVFX (500 mg/day until day 71), and left mastoidectomy was performed to drain the abscess from the middle ear cavity and mastoid. The left otorrhea resolved and the hematologic parameters improved within a few days after surgery. The subdural empyema had diminished two months after admission (Figure 3A). The size of the sigmoid sinus thrombosis decreased with treatment with edoxaban (Figure 3B), which was prescribed instead of continuous heparin after surgery until day 111. After antibiotic and anticoagulant treatment (Figure 3C), the lung nodule was considered a pulmonary septic embolus induced by sigmoid sinus thrombosis.

The final diagnosis was an otogenic variant of Lemierre’s syndrome. The patient was discharged from the hospital on day 50 after admission and remained symptom-free at 14 months of follow-up.

## Discussion

Lemierre's syndrome is typically initiated by pharyngitis or tonsillitis, followed by thrombophlebitis of the internal jugular vein and metastatic septic emboli. In this report, we describe a rare case of otogenic Lemierre's syndrome complicated by mastoiditis, subdural empyema, sigmoid sinus thrombosis, and septic pulmonary emboli. Otitis media with serious complications is rare in the modern era of antibiotic use. Although most of the patients with otitis media could be cured with antibiotics or surgical drainage, inflammation in the middle ear cavity can spread intracranially. Once a mastoid abscess secondary to otitis media ruptures through the middle cranial base, subdural empyema and suppurative thrombophlebitis of the sigmoid sinus vein, which is next to the mastoid, can occur, as in our case. To date, eight cases of otogenic Lemierre's syndrome, including this case, have been reported (Table 1) [2,5-10], including four males and three females (not specified in one patient).

**Table 1:** Summary of published cases of otitis media with septic pulmonary emboli N/A: Not applicable

Year	Author	Age/Sex	Microorganism	Aerobic or Anaerobic
1994	Allison et al. [5]	17/F	Proteus mirabilis	Anaerobic
1999	Stokroos et al. [6]	17/M	Fusobacterium necrophorum	Anaerobic
2002	Wong et al. [7]	12/F	N/A	N/A
2008	Monnier et al. [2]	N/A	Fusobacterium necrophorum	Anaerobic
2011	Lee et al. [8]	51/M	Enterobacter aerogenes	Anaerobic
2014	Turhal et al. [9]	30/M	Parvimonas micra	Anaerobic
2020	Bellazreg et al. [10]	45/M	N/A	N/A
2022	Present case	60/F	Trueperella bernardiae	Aerobic

The median patient age was 30 years (range: 12-60 years). All the patients were immunocompetent. The causative bacteria varied, and all the cases except this case were caused by anaerobic bacteria. To our knowledge, this is the first report of otogenic Lemierre's syndrome caused by an aerobic bacterial species.

T. bernardiae, a catalase-negative, Gram-positive aerobic bacillus, was the causative bacterium in this case. Although originally considered an opportunistic pathogen, T. bernardiae can cause severe infections [3,4,11-29]. Twenty-seven cases of T. bernardiae infection have been reported to date (Table 2).

**Table 2:** Summary of published cases of Trueperella bernardiae infection N/A: Not applicable

Year	Author	Age/Sex	Sites	Antibiotics	Outcome
1996	Ieven et al. [11]	69/M	Urinary tract infection	Amoxicillin-Clavulanate	Cured
1998	Adderson et al. [12]	19/F	Arthritis	Vancomycin, Cefotaxime, Clindamycin	Cured
1998	Lepargneur et al. [15]	75/M	Urinary tract infection	Netilmicin, Cefixime, Amoxicillin	Cured
2009	Bemer et al. [13]	63/M	Osteitis	Clindamycin, Fusidic acid	Cured
2009	Loïez et al. [14]	78/M	Prosthetic hip infection	Rifampicin, Ofloxacin	Cured
2010	Sirijatuphat et al. [16]	60/M	Urinary tract infection	Ceftazidime, Ceftriaxone, Clindamycin	Cured
2010	Clarke et al. [17]	62/F	Skin infection	Vancomycin, Aztreonam, Piperacillin-Tazobactam	Cured
2011	Weitzel et al. [18]	72/F	Sepsis	Ceftriaxone, Metronidazole, Amoxicillin-Clavulanate	Cured
2015	Schneider et al. [19]	45/M	Skin infection	Piperacillin-Tazobactam, Ciprofloxacin, Gentamicin, Amoxicillin	Cured
2015	Parha et al. [3]	68/M	Otitis media, Brain abscess	Gentamicin, Teicoplanin, Metronidazole, Ceftriaxone	N/A
2016	VanGorder et al. [20]	77/F	Skin infection	Trimethoprim-Sulfamethoxazole	Cured
2016	Rattes et al. [21]	24/F	Wound infection	Ppiperacillin-Tazobactam, Vanconmycin, Amoxicillin-clavulanate	Cured
2016	Gilarranz et al. [22]	73/F	Wound infection	Ciprofloxacin	Cured
2017	Cobo et al. [23]	69/F	Wound infection	Amoxicillin-Clavulanate	Cured
2017	Cobo et al. [23]	70/F	Wound infection	Amoxicillin-Clavulanate	Cured
2018	Lawrence et al. [24]	45/M	Wound infection	Cefuroxime, Metronidazole, Amoxicillin-Clavulanate	Cured
2018	Gowe et al. [25]	57/M	Olecranon bursitis	Ceftriaxone, Vanconmycin, Doxycyclin	Cured
2018	Calatrava et al. [26]	39/F	Breast abscess	Amoxicillin-clavulanate	Cured
2019	Pan et al. [4]	5/M	Otitis media, Brain abscess	Vancomycin, Cefepime, Metronidazole, Amoxicillin, Mastoidectomy	Cured
2019	Roh et al. [27]	83/F	Sepsis	Teicoplanin	Cured
2021	Tang et al. [28]	71/M	Prosthetic hip infection	Ceftriaxone, Cefadroxil	Cured
2022	Casale et al. [29]	78/F	Wound infection	Clindamycin, Metronidazole	Cured
2022	Present Case	60/F	Otitis media, Brain abscess	Amoxicillin, Meropenem, Levofloxacin	Cured

The median patient age was 69 years (range: 5-91 years), of whom 12 (44%) were male and 15 (56%) were female, and most patients were immunocompetent. Sepsis, urinary tract infection, and surgical wound infection were the most common types of infection, and there were three cases of otitis media with severe complications, including this case. T. bernardiae is generally sensitive to most antibiotics. In this case, the isolate was susceptible to ABPC, CEZ, IPM, and LZD. The patient was initially treated with ABPC, and the levels of inflammatory markers decreased. In cases of severe otitis media with mastoiditis, drainage of the abscess by mastoidectomy, in addition to antibiotic therapy, should be considered to ensure prompt remission. We were unable to perform mastoidectomy in the early phase because of the severe thrombocytopenia caused by disseminated intravascular coagulation; however, early surgical intervention to control mastoiditis is best, even for infections caused by aerobic bacteria, such as T. bernardiae.

## Conclusions

In conclusion, to our knowledge, this is the first case of otogenic Lemierre's syndrome caused by aerobic bacteria, T. bernardiae. The patient was successfully treated with antibiotics, mastoidectomy, and anticoagulants. Because otitis media with mastoiditis can induce rare but life-threatening complications, including sepsis, meningitis, sigmoid sinus thrombosis, subdural empyema, and septic pulmonary emboli, it should be considered a cause of severe infection and treated with appropriate antibiotics and surgery as early as possible.
